# On approximation and energy estimates for delta 6-convex functions

**DOI:** 10.1186/s13660-018-1637-7

**Published:** 2018-02-20

**Authors:** Muhammad Shoaib Saleem, Josip Pečarić, Nasir Rehman, Muhammad Wahab Khan, Muhammad Sajid Zahoor

**Affiliations:** 1 Department of Mathematics, University of Okara, Renala Khurd, Pakistan; 2 Faculty of Textile Technology, University of Zagreb, Zagreb, Croatia; 3 Department of Mathematics, Allam Iqbal Open University, Islamabad, Pakistan

**Keywords:** Convex function, Delta 6-convex functions, Mollification, Energy estimates

## Abstract

The smooth approximation and weighted energy estimates for delta 6-convex functions are derived in this research. Moreover, we conclude that if 6-convex functions are closed in uniform norm, then their third derivatives are closed in weighted $L^{2}$-norm.

## Introduction

In the recent decade, the study of convex functions and convex sets has developed rapidly because of its use in applied mathematics, specially in non-linear programming and optimization theory. Furthermore, the elegance shape and properties of a convex function develop interest in studying this branch of mathematics. But the classical definitions of convex function and convex set are not enough to overcome advanced applied problems. In the last few years, many efforts have been made on generalization of the notion of convexity to meet the hurdles in advanced optimization theory. Among many generalizations, some are quasi convex [1], pseudo convex [2], logarithmically convex [3], n-convex [4], delta convex [5], s-convex [6], h-convex [7], mid convex [8] and [9–14]. The function $f:I\rightarrow\mathbb{R}$ is said to be convex if $\forall x,y\in I$ and $\alpha,\beta\in[0,1] $ such that $\alpha +\beta=1$,

$$f(\alpha x + \beta y)\leq\alpha f(x) + \beta f(y) $$

holds. The function $f(x)\in C^{n}(I)$ is said to be n-convex if $f^{(n)}(x)\geq0$, $x\in I$. The weighted energy estimates for the convex function and 4-convex function are derived in [15] and [16]. These estimates are important in hedging strategies in finance [17]. Throughout the paper, we will use the following notations over *I*, where $I=[a,b]$.

$C(I)$
space of continuous functions over *I*.
$C^{6}(I)$
space of six times continuously differentiable functions on *I*.
$w(x)$
is the non-negative weight function which satisfies the following axiom:
  1.1 $$ \left . \textstyle\begin{array}{l@{\quad}l} w^{(\mathit{iv})}(x)\geq0 &\text{if } x \in I,\\ w{''}(x)\leq0 &\text{if } x \in I,\\ w(x)=w{'}(x)=w{''}(x)=w{'''}(x)=w^{(\mathit{iv})}(x)=w^{(v)}(x)=0 &\forall x\in\partial I . \end{array}\displaystyle \right \} $$

In the present paper, we deal with a delta 6-convex function. We derive some basic properties of the delta 6-convex function under certain conditions. Moreover, we approximate an arbitrary delta 6-convex function by smooth ones and derive weighted energy estimates for the derivative of delta 6-convex function.

### Definition 1.1

([18] Delta convex function)

The function $f:I\rightarrow\mathbb{R}$ is said to be delta convex function (or DC) over I if there exist continuous convex functions $f_{1}$ and $f_{2}$ on *I* such that

$$f=f_{1}-f_{2}. $$

### Definition 1.2

(Delta 6-convex function)

The function $f:I\rightarrow\mathbb{R}$ is said to be delta 6-convex function over I if there exist continuous 6-convex functions $f_{1}$ and $f_{2}$ on *I* such that

$$f=f_{1}-f_{2}. $$

The following proposition gives some basic properties of the delta 6-convex function.

### Proposition 1.3

*Let* *f* *and g be the two delta* 6-*convex functions*, *and let* $\alpha\geq 0$ *be real*. *Then*

(i) $f+g$ *is also a delta* 6-*convex function*.
(ii) *αf* *is also a delta* 6-*convex function*.
(iii) *Let* *g* *be increasing and* *f* *be a delta* 6-*convex function*, *then* $f\circ g$ *is also a delta* 6-*convex function*.

### Proof

The proof of the proposition is straightforward. □

## Approximation of a smooth delta 6-convex function and the statement of the main result

First we define the mollification of an arbitrary delta 6-convex function in $[a,b]$. The mollification of an arbitrary function is very well explained in the book by Evans [19]. Let $f(x)$ be an arbitrary delta 6-convex, 4-convex, and also 2-convex function. Then, by the property of the differentiability of the 6-convex, 4-convex, and 2-convex functions, $f \in C^{3}[a,b]$. Let $\theta _{\epsilon}\in C^{\infty}(\mathbb{R})$ have support on the interval $I_{\epsilon}=I (x_{0},r_{\epsilon})$. The *θ* is called approximation identity or mollifier. Take

$$ \theta_{\epsilon}(x)=\left \{ \textstyle\begin{array}{l@{\quad}l} c \exp{\frac{1}{x^{2}-1}}, & |x|< 1,\\ 0,& |x|\geq1, \end{array}\displaystyle \right . $$

where *c* is a constant such that

$$\int_{\mathbb{R}}\theta_{\epsilon}(x)\,dx=1. $$

Now, using $\theta_{\epsilon}$ as a kernel, we define the convolution of *f* as follows:

$$f_{\epsilon}(x)= \int_{\mathbb{R}} f(x-y)\theta_{\epsilon}(y)\,dy= \int _{\mathbb{R}} f(y)\theta_{\epsilon}(x-y)\,dy. $$

Since $\theta\epsilon\in C^{\infty}(\mathbb{R})$, so $f_{\epsilon}\in C^{\infty}(\mathbb{R})$.

If *f* is continuous, then $f_{\epsilon}$ converges uniformly to *f* in any compact subset $K\subseteq I$,

$$\vert {f}_{\epsilon}-f \vert \underset{\epsilon\to 0}{ \longrightarrow}0, $$

if $\epsilon=\frac{1}{m}$ then $\vert f_{m}-f \vert \underset{m\to \infty}{\longrightarrow}0$.

Let *f* be a delta 2-convex function, 4-convex function, and 6-convex function. We have to show that $f_{\epsilon}$ is also a delta 2-convex, delta 4-convex, and delta 6-convex function.

So,

$$ \begin{gathered}\begin{aligned}f_{\epsilon}(x)&= \int_{\mathbb{R}} f(x-y)\theta_{\epsilon}(y)\,dy \\ &= \int_{\mathbb{R}} f_{1}(x-y)\theta_{\epsilon}(y)\,dy- \int_{\mathbb {R}} f_{2}(x-y)\theta_{\epsilon}(y)\,dy,\end{aligned} \\ f_{\epsilon}(x)=f_{1,\epsilon}(x)-f_{2,\epsilon}(x).\end{gathered} $$

It is sufficient to prove that each $f_{i,\epsilon}(x)$ is convex, 2-convex, 4-convex, and 6-convex, where $i=1,2$.

Since

$$ \begin{aligned}f_{i,\epsilon} \bigl(\lambda x_{1}+(1- \lambda)x_{2} \bigr)&= \int _{\mathbb{R}} f_{i} \bigl(\lambda x_{1}+(1-\lambda)x_{2} -y \bigr)\theta _{\epsilon}(y)\,dy \\ & = \int_{\mathbb{R}} f_{i} \bigl(\lambda(x_{1}-y)+(1- \lambda) (x_{2}-y) \bigr)\theta_{\epsilon}(y)\,dy \\ & \leq \int_{\mathbb{R}} \bigl[\lambda f_{i}(x_{1}-y)+(1- \lambda )f_{i}(x_{2}-y) \bigr]\theta_{\epsilon}(y)\,dy \\ & = \int_{\mathbb{R}}\lambda f_{i}(x_{1}-y)\theta \epsilon y\,dy+ \int_{\mathbb{R}}(1-\lambda)f_{i}(x_{2}-y) \theta\epsilon(y)\,dy \\ & =\lambda f_{i,\epsilon}(x_{1})+(1-\lambda)f_{i,\epsilon}(x_{2}),\end{aligned} $$

so, $f_{i,\epsilon}$, $i=1,2$, is convex, which gives $f_{\epsilon}$ is a delta convex function. Similarly, the delta 2-convexity of $f^{(2)}$ and $f^{(4)}$ gives delta 4-convexity and 6-convexity of $f_{\epsilon}$. Now we give the statement of our main theorem.

### Theorem 2.1

*Let f*(*x*) *be an arbitrary delta* 6-*convex function over the interval I*. *Also*, *let f*(*x*) *be delta* 4-*convex and delta* 2-*convex*, *then the following holds*:

2.1 $$ \int_{I} \bigl\vert f{'''}(x) \bigr\vert ^{2} w(x)\,dx \leq \biggl[\frac{5}{4} \Vert f \Vert ^{2}_{L^{\infty}}+\frac{5}{2} \Vert f \Vert _{L^{\infty}} \bigl( \Vert f_{1} \Vert _{L^{\infty}}+ \Vert f_{2} \Vert _{L^{\infty}} \bigr) \biggr] \int_{I} \bigl\vert w^{(\mathit{vi})}(x) \bigr\vert \,dx, $$

*where* $w(x)$ *is a non*-*negative weight function which satisfies* (1.1). *And* $f_{1}$ *and* $f_{2}$ *are such that*

$$f=f_{2}-f_{1}. $$

### Remark 2.2

Let $f_{i}(x)$, $i=1,2$, be continuous arbitrary 6-convex functions over the interval *I*. Also, let $f_{i}(x)$, $i=1,2$, be 4-convex and 2-convex functions, then the following holds:

2.2 $$ \begin{aligned}[b] \int_{I} \bigl\vert f{'''}_{2}(x)-f_{1}{'''}(x) \bigr\vert ^{2} w(x)\,dx\leq {}&\biggl[\frac{5}{4} \Vert f_{2}-f_{1} \Vert ^{2}_{L^{\infty}}+ \frac {5}{2} \Vert f_{2}-f_{1} \Vert _{L^{\infty}} \bigl( \Vert f_{1} \Vert _{L^{\infty}}+ \Vert f_{2} \Vert _{L^{\infty}} \bigr) \biggr]\\ &\times \int_{I} \bigl\vert w^{(\mathit{vi})}(x) \bigr\vert \,dx,\end{aligned} $$

where $w(x)$ is a non-negative weight function which satisfies (1.1).

### Proof

By substituting $f=f_{2}-f_{1} $ in Theorem 2.1, we get the required result. □

## Some basic results and proof of the main result

Let $w(x)$ be the weight function which is non-negative, twice continuously differentiable, and satisfying

3.1 $$ w(a)=w(b)=0,\qquad w{'}(a)=w{'}(b)=0 $$

with $a\leq x\leq b$. We come to the following result of Hussain, Pecaric, and Shashiashvili [15].

### Lemma 3.1

*For the smooth convex function* $f(x)$ *and the non*-*negative weight function* $w(x)$ *defined on the interval* *I*, *satisfying* (3.1), *we have*

3.2 $$ \int_{I} \bigl(f{'}(x) \bigr)^{2}w(x) \,dx \leq \int_{I} \biggl[\frac{{(f(x))^{2}}}{2} +\sup_{x\in I} \bigl\vert f(x) \bigr\vert \bigl( f(x) \bigr) \biggr]\big|w{''}(x)\big|\,dx. $$

The results of 4-convex functions are established in [16].

### Lemma 3.2

*Let* $f(x)$ *be both* 4-*convex and* 2-*convex functions*. *Let* $w(x)$ *be the non*-*negative smooth weight function as defined in* (3.1) *and satisfying the condition*

3.3 $$ w{''}(x)\leq0\quad \forall x\in I \quad\textit{and}\quad w{'}(x)=w{''}(x)=w{'''}(x)=0 \quad\forall x\in\partial I. $$

*Then the following estimate holds*:

3.4 $$ \int_{I} \bigl(\big|f{''}(x)\big| \bigr)^{2}w(x) \,dx\leq \int_{I} \biggl(\frac{ ({{f(x)}} )^{2}}{2}-\sup_{x\in I}\big|f(x)\big| \bigl(f(x)\bigr) \biggr) w^{(\mathit{iv})}(x)\,dx. $$

We will start by the following theorem.

### Theorem 3.3

*Let* $f(x)\in C^{6}(I)$ *be a delta* 6-*convex function*. *Also f*(*x*) *is delta* 4-*convex as well as delta* 2-*convex*. *Then the following energy estimate is valid*:

3.5 $$ \begin{aligned}[b] \int_{I}\big|f_{2}{'''}(x)-f_{1}{'''}(x)\big|^{2}w(x) \,dx\leq {}&\biggl(\frac{5}{4}\| f_{2}-f_{1} \|^{2}_{{L^{\infty}}}+\frac{5}{2}\|f_{2}-f_{1} \|_{{L^{\infty}}} \bigl(\|f_{1}\|_{L^{\infty}}+\|f_{2}\|_{L^{\infty }} \bigr) \biggr)\\ &\times\big\| w^{(\mathit{vi})}(x)\big\| _{L^{1}},\end{aligned} $$

*where* $w(x)$ *is the weight function satisfying* (1.1).

To prove Theorem 3.3, we first prove the proposition.

### Proposition 3.4

*Let* $f, F \in C^{6}[a,b]$, *and* *F* *be* 6-*convex*, 4-*convex*, *as well as* 2-*convex function such that the condition*

3.6 $$ \left . \textstyle\begin{array}{l@{\quad}l} |f{''}(x)|\leq F{''}(x)& \forall x \in(a,b),\\ |f^{(\mathit{iv})}(x)|\leq F^{(\mathit{iv})}(x)& \forall x \in(a,b),\\ |f^{(\mathit{vi})}(x)|\leq F^{(\mathit{vi})}(x)& \forall x \in(a,b) \end{array}\displaystyle \right \} $$

*is fulfilled*. *Let* $w(x)$ *be a non*-*negative* 2-*concave*, 4-*convex weight function satisfying* (1.1), *then the following energy estimate is valid*:

3.7 $$ \int_{I}\big|f{'''}(x)\big|^{2}w(x)\,dx\leq \int_{I} \biggl(\frac {5(f(x))^{2}}{4}+\frac{5}{2}\|f \|_{L^{\infty}}F(x) \biggr)\big|w^{(\mathit{vi})}(x)\big|\,dx. $$

### Proof

Take

$$ \int_{I}\big|f{'''}(x)\big|^{2}w(x)\,dx= \int_{I}f{'''}(x) \bigl(f{'''}(x)w(x) \bigr)\,dx. $$

Using the integration by parts formula and making use of condition (1.1), we get

3.8 $$ \begin{aligned}[b] \int_{I}\big|f{'''}(x)\big|^{2}w(x)\,dx={}&{-} \int_{I} f{''}(x)f^{(\mathit{iv})}(x)w(x)\, dx \\ &- \int_{I} f{''}(x)f{'''}(x)w{'}(x) \,dx.\end{aligned} $$

Now take the first integral of (3.8) on the right-hand side. Using the integration by parts formula and condition (1.1), we have

3.9 $$ \begin{aligned}[b] ={}& \int_{I} f{'}(x)f^{(v)}(x)w(x)\,dx+ \int_{I} f{'}(x)f^{(\mathit{iv})}(x)w{'}(x) \,dx \\ &- \int_{I} f{''}(x)f{'''}(x)w{'}(x) \,dx.\end{aligned} $$

Now take the first and the second integrals on the right-hand side of the latter expression. Using the integration by parts formula and making use of condition (1.1), we get

3.10 $$ \begin{aligned}[b] ={}&{-} \int_{I} f(x)f^{(\mathit{vi})}(x)w(x)\,dx- \int_{I} f(x)f^{(v)}(x)w{'}(x)\, dx- \int_{I} f(x)f^{(v)}(x)w{'}(x)\,dx \\ &- \int_{I} f(x)f^{(\mathit{iv})}(x)w{''}(x)\,dx- \int_{I} f{''}(x)f{'''}(x)w{'}(x) \,dx.\end{aligned} $$

Proceeding in the similar way and using condition (1.1) and the definition of weight function, we obtain

3.11 $$ \begin{aligned}[b] ={}&{-} \int_{I} f(x)f^{(\mathit{vi})}(x)w(x)\,dx+ \int_{I} f(x)f^{(\mathit{iv})}(x)w{''}(x)\, dx \\ &+\frac{7}{2} \int_{I} \bigl(f{''}(x) \bigr)^{2}w{''}(x) \,dx- \int_{I} \bigl(f{'}(x) \bigr)^{2}w^{(\mathit{iv})}(x) \,dx.\end{aligned} $$

Now we take

$$\int_{I} \bigl(f{''}(x) \bigr)^{2}w{''}(x) \,dx. $$

Using Theorem 2.1 from [16], we have

3.12 $$ \begin{aligned}[b] \int_{I} \bigl(f{''}(x) \bigr)^{2}w{''}(x) \,dx={}& \int_{I} f(x)f^{(\mathit{iv})}(x)w{''}(x)\,dx - 2 \int_{I} f(x)f{''}(x)w^{(\mathit{iv})}(x)\, dx \\ &+\frac{1}{2} \int_{I} f^{2}(x)w^{(\mathit{vi})}(x)\,dx.\end{aligned} $$

Now, using (2.6) of [15], we have

3.13 $$ \begin{aligned}[b] \int_{I} \bigl(f{'}(x) \bigr)^{2} w^{(\mathit{iv})}(x)\,dx ={}&\frac{1}{2} \int_{I} f^{2}(x) w^{(\mathit{vi})}(x)\,dx \\ &- \int_{I} f(x)f{''}(x)w^{(\mathit{iv})}(x)\,dx.\end{aligned} $$

Substituting (3.12) and (3.13) in (3.11), we have

3.14 $$\begin{aligned} ={}&{-} \int_{I} f(x)f^{(\mathit{vi})}(x)w(x)\,dx + \frac{9}{2} \int_{I} f(x)f^{(\mathit{iv})}(x)w{''}(x)\,dx \\ &-6 \int_{I} f(x)f{''}(x)w^{(\mathit{iv})}(x)\,dx+ \frac{5}{4} \int_{I} f^{2}(x)w^{(\mathit{vi})}(x)\,dx \\\leq{}&\sup_{x\in I}\big|f(x)\big| \int_{I} \big|f^{(\mathit{vi})}(x)\big|\big|w(x)\big|\,dx + \frac{9}{2} \sup_{x\in I}\big|f(x)\big| \int_{I} \big|f^{(\mathit{iv})}(x)\big|\big|w{''}(x)\big|\,dx \\&+6 \sup_{x\in I}\big|f(x)\big| \int_{I} \big|f{''}(x)\big|\big|w^{(\mathit{iv})}(x)\big|\,dx + \frac {5}{4} \int_{I} f^{2}(x)\big|w^{(\mathit{vi})}\big|(x)\,dx. \end{aligned}$$

Here,

$$\begin{gathered} \big|f{''}(x)\big|\leq F{''}(x), \\\big|f^{(\mathit{iv})}(x)\big|\leq F^{(\mathit{iv})}(x), \\\big|f^{(\mathit{vi})}(x)\big|\leq F^{(\mathit{vi})}(x), \\w{''}(x)\leq0, \\w^{(\mathit{iv})}(x)\geq0.\end{gathered} $$

Using the above conditions, we obtain

$$\begin{aligned} \leq{}&\sup_{x\in I}\big|f(x)\big| \int_{I} F^{(\mathit{vi})}(x)w(x)\,dx - \frac{9}{2} \sup _{x\in I} \int_{I} F^{(\mathit{iv})}(x)w{''}(x)\,dx \\&+6 \sup_{x\in I}\big|f(x)\big| \int_{I} F{''}(x)w^{(\mathit{iv})}(x)\,dx + \frac {5}{4} \int_{I} f^{2}(x)\big|w^{(\mathit{vi})}\big|(x)\,dx.\end{aligned} $$

Using the integration by parts formula, we obtain

$$\begin{aligned} \leq{}&\sup_{x\in I}\big|f(x)\big| \int_{I} F(x)w^{(\mathit{vi})}(x)\,dx - \frac{9}{2} \sup _{x\in I} \int_{I} F(x)w^{(\mathit{vi})}(x)\,dx \\&+6 \sup_{x\in I}\big|f(x)\big| \int_{I} F(x)w^{(\mathit{vi})}(x)\,dx + \frac{5}{4} \int _{I} f^{2}(x)\big|w^{(\mathit{vi})}\big|(x)\,dx.\end{aligned} $$

Hence,

3.15 $$\begin{aligned}& \begin{aligned}[b] \int_{I} \bigl(f{'''}(x) \bigr)^{2}w(x) \,dx\leq{}& \biggl[\sup_{x\in I}\big|f(x)\big| \int_{I} F(x)-\frac{9}{2}\sup_{x\in I}\big|f(x)\big| \int_{I} F(x) \\ &+6\sup_{x\in I}\big|f(x)\big| \int_{I} F(x)+\frac{5}{4} \int_{I} \bigl(f^{2}(x) \bigr) \biggr]w^{(\mathit{vi})}(x) \,dx,\end{aligned} \end{aligned}$$

3.16 $$\begin{aligned}& \int_{I} \bigl(f{'''}(x) \bigr)^{2}w(x)d \leq \int_{I} \biggl(\frac {5(F(x))^{2}}{4}+\frac{5}{2}\sup _{x\in I}\big|F(x)\big|\times \bigl(F(x) \bigr) \biggr)w^{(\mathit{vi})}(x) \end{aligned}$$

as the required proof. □

The following weighted energy inequality for the smooth 6-convex function can be obtained simply by taking $F=f$ in (3.16), where $f\in C^{6}[a,b]$ and *f* and *w* satisfy the conditions of the last theorem. Then we have

3.17 $$ \int_{I}\big|f{'''}(x)\big|^{2}w(x)\,dx\leq \int_{I} \biggl(\frac {5(f(x))^{2}}{4}+\frac{5}{2}\|f \|_{L^{\infty}}f(x) \biggr)w^{(\mathit{vi})}(x)\,dx. $$

The next result describes the energy estimate for the difference of two 6-convex functions.

### Proof of Theorem 3.3

Take $f=f_{2}-f_{1}$ and $F=f_{1}+f_{2}$ in Proposition (3.4) to get

3.18 $$ \begin{aligned}[b] \int_{I}\big|f_{2}{'''}(x)-f_{1}{'''}(x)\big|^{2}w(x) \,dx\leq{}& \int_{I} \biggl[\frac {5}{4}\big\| f_{2}(x)-f_{1}(x) \big\| ^{2}_{L^{\infty}} \\ &+\frac{5}{2}\|f_{2}-f_{1} \| _{{L^{\infty}}}\bigl(\|f_{2}\|_{L^{\infty}}+\| f_{1}\|_{L^{\infty}} \bigr) \biggr]w^{(\mathit{vi})}(x)\,dx.\end{aligned} $$

 □

We conclude the section with the following remark.

### Remark 3.5

Let $f_{1}$, $f_{2}$, and $w(x)$ be the same as in the latter theorem. Then, by using Holder’s inequality, we have

3.19 $$ \int_{I}\big|f_{2}{'''}(x)-f_{1}{'''}(x)\big|^{2}w(x) \,dx\leq\|\widetilde{f}\| _{L^{p}}\big\| w^{(\mathit{vi})}(x)\big\| _{L^{q}}, $$

where $\frac{1}{p}+\frac{1}{q}=1$ and

3.20 $$ \widetilde{f}(x)=\frac{5 (f_{2}(x)-f_{1}(x) )^{2}}{4}+\frac {5}{2}\|f_{2}-f_{1} \|_{L^{\infty}} \bigl(f_{1}(x)+f_{2}(x) \bigr). $$

### Proof of Theorem 2.1

For the continuous arbitrary 6-convex functions $f_{i}(x)$, $i=1,2$, consider the smooth approximation $f_{m,i}(x)$, $i=1,2$.

For the interval $I_{k+l}$, there exists an integer $m_{k+l}$ such that $f_{m,i}(x)$ converges uniformly to $f_{i}(x)$, $i=1,2$, and also $f_{m,i}(x)$ is smooth for $m\geq m_{k+l}$.

Now, writing inequality (3.18) for the functions $f_{m,1}$ and $f_{m,2}$ over the interval $I_{k+l}$, we get

3.21 $$ \begin{aligned}[b] \int_{I_{k+l}} \bigl\vert f_{m,2}{'''}(x)-f_{m,1}{'''}(x) \bigr\vert ^{2} w(x)\, dx\leq{}& c_{k+l} \biggl[ \frac{5}{4} \Vert f_{m,2}-f_{m,1} \Vert ^{2}_{L^{\infty}} \\ & +\frac{5}{2} \Vert f_{m,2}-f_{m,1} \Vert _{L^{\infty}} \bigl( \Vert f_{m,1} \Vert _{L^{\infty}} + \Vert f_{m,2} \Vert _{L^{\infty}} \bigr) \biggr],\end{aligned} $$

where $c_{k+l}=\int_{I_{k+l}} \vert w^{(\mathit{vi})}(x) \vert \,dx$.

Now, taking limit $m\rightarrow\infty$, we obtain

3.22 $$ \begin{aligned}[b] \int_{I_{k+l}} \bigl\vert f_{2}{'''}(x)-f_{1}{'''}(x) \bigr\vert ^{2} w(x)\,dx \leq{}& c_{k+l} \biggl[ \frac{5}{4} \Vert f_{2}-f_{1} \Vert ^{2}_{L^{\infty}(I_{k+l})} \\ &+\frac{5}{2} \Vert f_{2}-f_{1} \Vert _{L^{\infty}(I_{k+l})} \bigl( \Vert f_{1} \Vert _{L^{\infty}(I_{k+l})}+ \Vert f_{2} \Vert _{L^{\infty}(I_{k+l})} \bigr) \biggr].\end{aligned} $$

Now, writing the left-hand integral for the smaller interval $I_{k} \subset I_{k+l}$ and also taking limit $l \rightarrow\infty$, we obtain

3.23 $$ \begin{aligned}[b] \int_{I_{k}} \bigl\vert f_{2}{'''}(x)-f_{1}{'''}(x) \bigr\vert ^{2} w(x)\,dx \leq{}& c_{\infty} \biggl[ \frac{5}{4} \Vert f_{2}-f_{1} \Vert ^{2}_{L^{\infty}(I)} \\ & +\frac{5}{2} \Vert f_{2}-f_{1} \Vert _{L^{\infty}(I)} \bigl( \Vert f_{1} \Vert _{L^{\infty}(I)} + \Vert f_{2} \Vert _{L^{\infty}(I)} \bigr) \biggr].\end{aligned} $$

Since we have

$$\int_{I} \bigl\vert f_{i}{'''}(x) \bigr\vert ^{2}w(x)\,dx< \infty, \quad i=1,2, $$

taking limit as $k\rightarrow\infty$, we obtain the required result (2.1). □

## Conclusion

From the result (2.2) we conclude that, if 6-convex functions are closed in uniform norms, then their third derivatives are also closed in weighted $L^{2}$-norm.

## References

[1] Definetti B. (1949). Sulla stratificazioni convesse. Ann. Math. Pures Appl..

[2] Mangasarian O.L. (1965). Pseudo-convex functions. SIAM J. Control.

[3] Pečarić J., Proschan F., Tong Y.L. (1992). Convex Functions, Partial Ordering and Statistical Applications.

[4] Bullen P.S. (1971). A criterion for n-convexity. Pac. J. Math..

[5] Rajba T. (2015). On strong delta-convexity and Hermite–Hadamard type inequalities for delta-convex functions of higher order. Math. Inequal. Appl..

[6] Ozdemir M.E., Yildiz C., Akdemir A.O., Set E. (2013). On some inequalities for s-convex functions and applications. J. Inequal. Appl..

[7] Varosanec S. (2007). On h-convexity. J. Math. Anal. Appl..

[8] Jensen J.L.W.V. (1905). On konvexe funktioner oguligheder mellem middlvaerdier. Nyt Tidsskr. Math. B..

[9] Abramovich S. (2013). Convexity, sub-additivity and generalized Jensen’s inequality. Ann. Funct. Anal..

[10] Aleman A. (1985). On some generalizations of convex sets and convex functions. Rev. Anal. Numér. Théor. Approx..

[11] Anastassiou G.A. (2013). Basic and s-convexity Ostrowski and Gruss type inequalities involving several functions. Commun. Appl. Anal..

[12] Kuroiwa D. (1996). Convexity for set-valued maps. Appl. Math. Lett..

[13] Luc D.T. (1989). Theory of Vector Optimization.

[14] Set E., Ozdemir M.E., Dragomir S.S. (2010). On Hadamard-type inequalities involving several kinds of convexity. J. Inequal. Appl..

[15] Hussain S., Pečarić J., Shashiashvili M. (2008). The weighted square integral inequalities for the first derivative of the function of a real variable. J. Inequal. Appl..

[16] Pečarić J., Saleem S., Ahmed I., Ahmed N. (2017). Weighted integral inequalities for the second derivative of 4-convex function. J. Math. Inequal..

[17] Hussain S., Rehman N. (2012). Estimate for the discrete time hedging error of the American option on a dividend paying stock. Math. Inequal. Appl..

[18] Busemann H., Feller W. (1936). Krummungseigenschaften Konvexer Flachen. Acta Math..

[19] Evans L.C. (1998). Partial Differential Equations.

